# Bacterial Semiochemicals and Transkingdom Interactions with Insects and Plants

**DOI:** 10.3390/insects10120441

**Published:** 2019-12-08

**Authors:** Matteo Calcagnile, Salvatore Maurizio Tredici, Adelfia Talà, Pietro Alifano

**Affiliations:** Department of Biological and Environmental Sciences and Technologies, University of Salento, Via Prov.le Lecce-Monteroni, 73100 Lecce, Italy; matteo.calcagnile@unisalento.it (M.C.); maurizio.tredici@unisalento.it (S.M.T.); adelfia.tala@unisalento.it (A.T.)

**Keywords:** microbiota, microbiome, symbiosis, insect semiochemicals, bacterial metabolism

## Abstract

A peculiar feature of all living beings is their capability to communicate. With the discovery of the quorum sensing phenomenon in bioluminescent bacteria in the late 1960s, it became clear that intraspecies and interspecies communications and social behaviors also occur in simple microorganisms such as bacteria. However, at that time, it was difficult to imagine how such small organisms—invisible to the naked eye—could influence the behavior and wellbeing of the larger, more complex and visible organisms they colonize. Now that we know this information, the challenge is to identify the myriad of bacterial chemical signals and communication networks that regulate the life of what can be defined, in a whole, as a meta-organism. In this review, we described the transkingdom crosstalk between bacteria, insects, and plants from an ecological perspective, providing some paradigmatic examples. Second, we reviewed what is known about the genetic and biochemical bases of the bacterial chemical communication with other organisms and how explore the semiochemical potential of a bacterium can be explored. Finally, we illustrated how bacterial semiochemicals managing the transkingdom communication may be exploited from a biotechnological point of view.

## 1. Introduction

The life of every living being takes place in a dynamic network of relationships with other organisms.

Among the interspecific interactions, those occurring between micro- and macro-organisms have attracted increasing scientific interest and public attention, thus unveiling new and unexpected roles of bugs as beneficial modulators of many biological processes in plant and animal hosts, extending and enhancing their adaptive capabilities.

It is understood that rhizosphere symbiotic microorganisms exert fundamental nutritional and protective effects on different species of plants, strongly contributing to their ecological success [1]. Mycorrhizal fungi provide plants with water and mineral nutrients collected in the soil and counteract soilborne pathogens [2]. N_2_-fixing bacteria fulfill the nitrogen requirement of several plants in nitrogen-depleted soils [3]. Plant growth-promoting rhizobacteria (PGPR) stimulate plant growth by synthesizing phytohormones and vitamins and exert protective effects on plants against both abiotic and biotic stresses through a variety of well-documented mechanisms [4]. PGPR counteract the water stress by producing exopolysaccharides that alter the soil texture and allow more effective root penetration in the soil. PGPR also sequester and/or degrade toxic compounds, such as heavy metals or xenobiotic aromatic compounds. Moreover, they limit the growth of pathogenic microorganisms and/or protect the plants from their aggression using a variety of mechanisms, including iron limitation by siderophores, the production of antibiotics and extracellular enzymes that attack the microbial cell wall, and elicitation of the induced systemic resistance (ISR) [5]. In turn, in all cases, the host plants provide the symbiotic microorganisms with a protected ecological niche and rhizospheric organic carbon [6]. In insects, symbiotic microorganisms, mostly bacteria, modulate many processes including nutrition, development, immune system, and social and sexual behaviors, making the conquest of new ecological niches possible and, ultimately, promoting the insect evolution [7].

Interestingly, in terrestrial ecosystems, symbiotic microorganisms oversee plant–insect interactions, sometimes according to rather conserved patterns. In general, PGPR exert negative effects on the abundance and activity of herbivore insects, likely due to the priming of primary defenses in the plant [8,9]. In contrast, arbuscular mycorrhizal fungi (AMF) have positive effects on sap-sucking herbivore insects. This is likely due to positive effects of symbiotic fungi on the nutrient level in the phloem [8,9]. AMF effects on chewing herbivore insects are, instead, generally neutral. However, when present, the effects are beneficial for specialist chewing herbivores and detrimental for those of the general kind. Furthermore, AMF generally exert negative effects on natural plant enemies [10]. In the establishment of specialized plant–insect interactions, such as in the case of the interaction between *Mentha aquatica* and *Chrysolina herbacea* [11], an aspect of great interest is represented by the dynamics of plant- and insect-associated microbial communities. This also applies to the role of bacterial volatile organic compounds (BVOCs) in regulating the crosstalk between plants and generalist herbivores and in protecting plants from generalist herbivores.

Indeed, an essential aspect of the interspecific interaction is that of communication that triggers and controls the specific biological responses that underlie the symbiosis. Recently, scientific literature has attributed an increasingly important role to the microbial volatile organic compounds (MVOCs) and, in particular, of BVOCs in the chemical communication between microorganisms, plants, and insects [12,13]. There are a number of examples illustrating how secondary metabolites produced by symbiotic bacteria may act as intraspecific, interspecific, and mating signals in insects affecting their sexual and social behavior, and how bacterial secondary metabolism and plant secondary metabolism may be functionally interconnected between them to produce chemical signals that may be active toward specific insect populations [14,15].

Bacteria play surprising roles in presocial and eusocial insect intraspecies communication. Presocial skills, which are defined as simple phenomena mediated by pheromones, are very diffuse in many insect orders. Specific BVOCs, which are mostly produced by gut bacteria, work as multifunctional pheromones in presocial insect communication. More sophisticated mechanisms underlie eusocial insect communication, which supports reproduction, the development from egg to adult, and influences the behaviors during all life stages. Hymenoptera, particularly ants, bees, and wasps, are the most well-known examples of eusocial insects. These insects developed complex social skills during evolution, implementing a complex network between individuals and forming nests. Cuticular hydrocarbons (CHCs) play a key role in the social insect communication, and there is some evidence that the social insect microbiota may be involved in CHC metabolism [16]. As well as in presocial insect communication, MVOCs, in particular BVOCs produced by gut bacteria, are involved in eusocial insect and sexual communication. Also, positive (mutualism) or negative (parasitism) interspecies relationships are established using mechanisms and compounds (BVOCs) related to social communication in insects [17].

Elucidating the biological role, the biochemistry and regulation of the chemical signals produced by microorganisms is not only essential to better understanding the ecological significance of transkingdom interactions involving microorganisms, plants, and insects, but it is also instrumental to the development of biotechnological devices. This tool is imperative for understanding the biological struggle for more sustainable agriculture and to deal with the effects of the climate changes on plant–insect ecosystems, which inevitably involve changes in microbial community dynamics with a far greater knowledge [18].

## 2. Interactions and Chemical Crosstalk between Microorganisms, Plants, and Insects: Ecological Models and Study Cases

Transkingdom interactions involving microorganisms, plants, and insects are generally complex, multifaceted, and arduous to analyze. They involve multiple organisms at different macro- and micro-scale levels, thus, taking transkingdom interactions beyond the classic schemes exemplified by the major types of biological interactions involving pairs of organisms and often causing them to be conditional and affected by abiotic environmental parameters [19]. Consequently, the chemical crosstalk that oversees transkingdom interactions is sophisticated and hard to decipher from an ecological point of view. Another concern when studying the role of microbial semiochemicals in transkingdom interactions is relating the chemical signals that a microorganism can produce at a micro-scale level in a natural environment to a biological response in a macro-organism [20]. Therefore, the setup of appropriate ecological models to consolidate results is strongly encouraged. Below, we describe several ecological models suitable for studying the interactions between microorganisms, plants, and insects, along with the established or hypothesized role of compounds of microbial origin in the transkingdom communication. Here, the role of bacteria and products of their metabolism in intraspecies presocial and eusocial insect communication, in sexual communication, and in interspecies communication supporting mutualism or parasitism is illustrated.

### 2.1. Bacteria and Presocial Insect Communication

**Figure 1:**
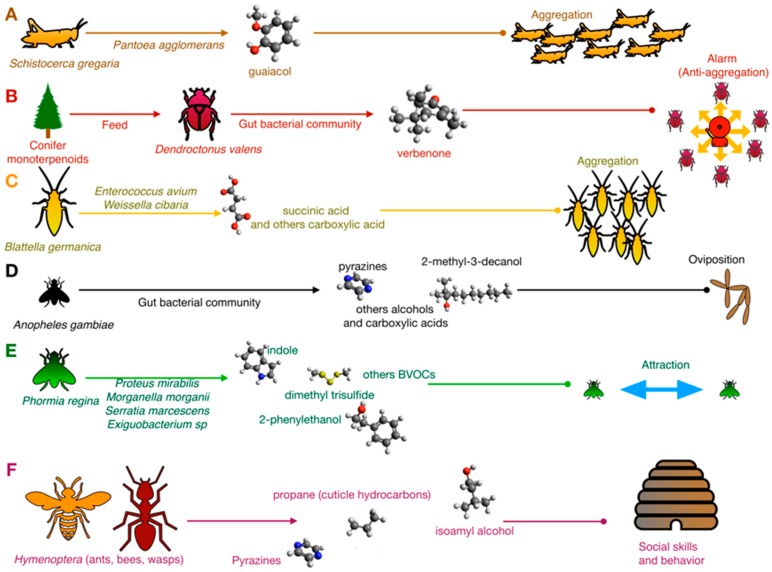
Bacteria and insect communication. In *S. gregaria* (**A**), the bacterium *P. agglomerans* produces guaiacol, which acts as an aggregation signal. In *D. valens* (**B**), bacterial community convert conifer monoterpenoids in verbenone, a multifunctional pheromone. In *B. germanica* (**C**), the aggregation is stimulated by succinic acid and other acids produced by gut bacteria. In *A. gambiae* (**D**), pyrazines, carboxylic acids, and alcohols stimulate oviposition. In *P. regina* (**E**), bacterial volatile organic compounds (BVOCs) stimulate attraction between individuals. In eusocial insects (**F**) (Hymenoptera), social and behavioral skills are managed by pyrazines, cuticular hydrocarbons, and other semiochemicals.

The bacterial contribution to chemical crosstalk was first investigated in simple phenomena such as attraction/cohesion processes or sexual behavior. For example, Dillon and colleagues [21,22] demonstrated that in the desert locust *Schistocerca gregaria* (Orthoptera, Acrididae), the aggregation pheromone guaiacol was produced in vitro by *Pantoea aggolomerans* isolated from the insect gut using vanillic acid introduced by diet as a substrate (Figure 1).

Microbial biotransformations are, indeed, rather common in insects. In the bark beetle *Dendroctonus valens* (Coleoptera, Curculionidae), the most important mortality agent in the coniferous forest, the gut microbiota transforms toxic monoterpenoids of conifers into the multifunctional pheromone verbenone [23,24]. In *D. valens*, the verbenone works as an aggregation pheromone at a low concentration, while it is an alarm (anti-aggregation) pheromone at a high concentration. Thus, it has been proposed for coniferous forest management. Gut bacteria transform *cis*-verbenol, a toxic precursor for bacteria, to verbenone. It subsequently benefits from this conversion due to the reduced toxicity of verbenone. At the same time, by producing and modulating the bark beetle ambivalent pheromone, they play a key role in controlling *D. valens* population density (Figure 1). Specific groups of terpene-metabolizing bacteria that are associated with monoterpene tolerance were described in different *Dendroctonus* species. In *D. valens*, the terpene-metabolizing bacteria were almost equally represented by both Gram-positive (Actinobacteria, Bacilli) and Gram-negative (Gamma-Proteobacteria) species, while in *D. ponderosae*, the Gram-positive species were predominant. Compared to the bacteria isolated from *D. valens*, the bacteria from *D. ponderosae* were not able to grow using α-pinene and 3-carene as carbon substrate. They were therefore inhibited by different monoterpenes [24], suggesting that a reduction in Gammaproteobacteria may affect their tolerance to monoterpenes. A different study reported that several *Serratia* strains from *D. ponderosae* were able to degrade monoterpenes but not α-pinene that was, instead, degraded by *Rahnella*. On the other hand, *Serratia* and *Brevundimonas* were able to reduce diterpenes [25]. Compounds and strains involved in these systems are reported in Table S1 of Supplementary Materials.

The upmost importance of bacteria in insect aggregation was also demonstrated in the German cockroach *Blattella germanica* (Blattodea, Blattellidae). This cockroach is a major pest of the built environment, where it can acquire and transmit pathogens and produce allergens that cause disease in humans. Wada-Katsumata and coworkers [26] demonstrated that the feces of normal cockroaches emitted volatile carboxylic acids (VCAs) that elicited insect aggregation, whereas bacteria-free feces contained few VCAs and were relatively unattractive. Among bacteria, *Enterococcus avium* and *Weissella cibaria* were shown to produce aggregation BVOCs 3-isovaleric acid and valeric, succinic acid, benzoic acid, and phenylacetic acid [26] (Figure 1).

A similar mechanism was also demonstrated in the black blow fly *Phormia regina* (Diptera, Calliphoridae), in which bacteria of the species *Proteus mirabilis*, *Morganella morganii*, *Serratia marcescens,* and *Exiguobacterium* spp. emit strain-specific BVOCs (Table S1) which work as attraction signals [27] (Figure 1).

Many studies were focused on aphids (Rhynchota, Aphidiae), a common group of plant parasites that live in a colony and feed on plant sap releasing honeydew, a sugar-rich, dense liquid used by Hymenoptera as a feed source. Different semiochemicals dealing with intraspecies communication are present in honeydew, including the *E*-*β*-farnesene and a number of BVOCs (Table S1). *E*-*β*-farnesene is an alarm pheromone [28,29,30] and is a key component of communication. Sixteen out of 26 aphid species emit this compound as the only or among the most common volatiles [31].

In insects, BVOCs are also signals for oviposition (Figure 1). In *Anopheles gambiae* (Diptera, Culicidae), an African mosquito considered one of the most effective vectors of malaria, an inoculum of gut bacteria stimulated oviposition through semiochemicals 2-methyl-3-decanol 24, 3-methyl-1-butanol 21, 2-phenylethanol 11, indole 9, 3-methylbutanoic acid 25, diisopropylpyrazine 26, 2,5-diisopropylpyrazine 27, isopropyl-secbutylpyrazine 28, isopropyl-isobutylpyrazine 29, phenylmethanol 30, and 2-phenylethanol 31 [32,33] (Table S1). Other behavioral responses of this mosquito are based on semiochemicals signature produced by the microbiota: BVOCs released by human microbiota on the skin surface drive the choice of the host, influencing the diffusion of malaria directly [34]. *Staphylococcus epidermidis* releases various BVOCs that influence this phenomenon, such as 3-methyl butanal, 2-methyl butanal and 2-methylbutanoic acid, and 3-methyl-1-butanol and 3-methylbutanoic acid [35]. A similar phenomenon occurs in yellow fever vector *Aedesa egypti*, in which the relationship between the attraction of adult female individuals and quorum sensing regulation in *S. epidermidis* was investigated [36].

### 2.2. Bacteria and Eusocial Insect Communication

In social nest-living insects, BVOCs regulate social behaviors. For example, in *Atta sexdens* (Hymenoptera, Formicidae), 2,5-dimethylpyrazine and 3-ethyl-2,5-dimethylpyrazine were identified in poison gland secretion and work as powerful trail pheromones (Figure 1). In *A. sexdens* var. rubropilosa (Hymenoptera, Formicidae), *Serratia marcences*, a common endosymbiotic strain in insects, is involved in pyrazines biosynthesis starting from L-threonine as a precursor [37]. In addition to bacteria, yeast species also contribute to semiochemical communication in Hymenoptera. In North American yellowjacket wasps (Hymenoptera, Vespidae), two species of fungi produce VOCs that attract conspecific individuals. *Hanseniaspora uvarum* and *Lachancea thermotolerans* were attractive to three species of yellowjacket wasps (*V. pensylvanica*, *V. germanica*, and *V. alascensis*) when grown on grape juice-infused yeast peptone dextrose (YPD) agar [38]. Gas chromatography–mass spectrometry analysis revealed that ethyl acetate (only *H. uvarum*), phenylethyl acetate, isoamyl acetate, 2-phenylethyl alcohol, isoamyl alcohol, and 2-methyl-butanol were the most abundant headspace volatiles produced by these yeasts. This information was instrumental to formulate a synthetic blend that was proven to be effective in attracting *V. pensylvanica*, but not the other yellowjacket species [38].

Another important type of communication in social insects is based on chemical recognition of CHCs. In termite species, the aggressive response is common when a parasite or non-nestmate insect penetrates the nest. In *Reticulitermes speratus* (Isoptera, Rhinotermitidae), antibiotic feeding increases the mortality and aggression of test individuals [39]. Similar effects were obtained with the addition of extracts from gut bacteria of non-nestmate individuals [39]. The death or reduction of microbial symbionts also affects the fitness of individuals, showing a reduction in body size and in mechanical resistance of the exoskeleton formed by a thinner cuticle [40]. Direct evidence of CHC metabolism by gut bacteria was obtained in *Zootermopsis nevadensis* (Isoptera, Termopsidae) using radiolabeled succinate, a precursor of methyl-branched hydrocarbons, which was transformed by bacteria into propionate and incorporated in the cuticle as 5-methyl-heneicosane or 5,17-dimethylheneicosane by insect cells [41]. A specific analysis of the effects of gut microbiota on cuticular hydrocarbon metabolism in ants was performed by Teseo and colleagues [41] based on three point-of-view approaches. These experiments investigated the relationship between variation in bacterial community, differences in CHC profiling, and nestmate recognition. The authors manipulated the gut microbiota of *Acromyrmex echinatior* leaf-cutting ants (Hymenoptera, Formicidae) using tetracycline. Before partially restoring the microbiota through fecal droplet administration, they analyzed the relationship between microbiota structure and insect survival and found that survival was enhanced by endosymbionts. Moreover, they proved that antibiotic treatment modified the normal microbiota structure and affected the CHC chemical profile. Additionally, results showed that modified CHC chemical profile was significantly correlated with enhanced aggression and misrecognition. Variations in CHC levels were significant for 4-oxo-octanoic acid, 4-oxo-decanoic acid and in long-chain linear alkanes (n-C36 and n-C40), which decreased in antibiotic-treated insects compared to untreated controls [42].

Finally, in eusocial insects, the gut microbiota assemblage reflects the exposure to social interactions: Oral trophallaxis enhanced the development a healthy “core microbiota” in the bee. Gram negative bacteria, such as *Gilliamella apicola*, *Frischella perrara,* and *Snodgrassella alvi,* are acquired with either social interaction between young individuals, nurse/workers individuals, or those that have contact on hive surfaces. These studies have shown how the social contact may affect the normal gut microbiota in the virgin queen bee [43,44]. Other studies demonstrated that the *Apis* microbiota is caste-specific [45,46] and age-specific [47]. It is also affected by the environmental landscape [48]. Queens and workers harbor strikingly different gut microbiota, and the queen replacement affects the microbiota in workers [47]. These phenomena can explain that any nest or group of insects has a specific volatile profile, a specific microbiota, and a specific cuticular profile that acts as molecular signature and contributes not only at the intraspecific recognition, but also as social and behavior skills at intranest level.

### 2.3. Bacteria and Sexual Communication in Insects

Sexual recognition in the insect is another intriguing phenomenon influenced by bacteria. Specifically, the microbiota can influence reproduction with different mechanisms, such as sexual manipulation or pheromone and semiochemical manipulation. Sexual manipulators include bacteria of the genera *Wolbachia*, *Rickettsia*, *Cardinium*, *Spiroplasma*, and *Arsenophonus*. They affect reproduction in different ways: They may induce parthenogenesis, feminization, or male killing [49]. On the other hand, a number of bacteria and some viruses have the ability to influence the sexual behaviors.

In an interesting model, the Hz-2v virus was shown to enhance sexual pheromone production in *Helicoverpa zea* (Lepidoptera, Noctuidae) females that attracted more males than non-infected females, thereby facilitating virus transmission [50]. Meanwhile, the bacterium *Staphylococcus aureus* was shown to manipulate pheromone levels in male boll weevils thanks to the activity of Enterotoxin B [51].

In *Costelytra zealandica* (Coleoptera, Scarabaeidae), symbiont bacteria belonging to the species *Morganella morganii* isolated from colleterial glands produce phenol form tyrosine as a female sexual pheromone [52,53]. The CHC chemical profile can also regulate sexual interactions between ants: Male individuals of *Cardiocondyla obscurior* (Hymenoptera, Formicidae) genus mimic CHCs of females in sexual conflict [54,55], and an altered gut microbial community causes leaking of tyrosine precursor 4-hydroxyphenylpyruvate for cuticle formation. This precursor is provided by the *Westeberhardia* bacterial symbiont [56].

In the plant–insect systems formed by *Chrysolina herbacea* (Coleoptera, Chrysomelidae, Chrysomelinae) and *Mentha aquatica*, diet and sexual identity are key factors that drive sexual recognition between individuals [57]. *M. aquatica* adopt a constitutive defense system against herbivore based on terpenoids biosynthesis in glandular trichomes [58]. Some terpenoids regulate the attraction of insects: Pulegone, a major compound emitted by undamaged plants, is a powerful attractant for *C. herbacea*, while menthofuran works as a repelling agent produced by damaged plants [11]. GC–MS analysis of *M. aquatica* oils before and after the inoculum of bacterial consortia isolated from the insect gut revealed as BVOCs amount and type are linked to the sex of individuals [57]. In the microbial community from female individuals 3-nonanol, β-bourbonene, isopulegol, α-amorphene, and longipinanol were detected, while 3-nonanol and isopulegol were detected in the microbial community from males. Both communities could biotransform the essential oil by producing menthofuran, menthone, 1,8-cineole, limonene, and pulegone. Several of these compounds were previously described as pheromones in insects. For instance, in *Crematogaster sjostedti* (Hymenoptera, famiglia), 3-nonanol mediates alarm, defense response, and conspecific recognition [59]. In *Azteca chartifex* (Hymenoptera, famiglia), α-amorphene is detected in pygidial gland secretions, while β-bourbonene is a pheromone in *Euceraphis punctipennis* (Rhynchota, Aphidiae) [31,32,33,34,35,36,37,38,39,40,41,42,43,44,45,46,47,48,49,50,51,52,53,54,55,56,57,58,59,60].

### 2.4. Bacteria and Interspecies Insect Communication

As described before, microorganisms may influence chemical signaling with BVOC emission or CHC metabolism in insect communities. In a number of mutualistic symbioses, as well as in some parasitic interactions between insect species, the acquisition and exchange of microbial strains can induce a phenomenon of chemical camouflage that reduces the aggressiveness between non-nestamate insects [61].

In literature, many reported observations of mutualist relationships were focused on ants for different reasons: (I) Simple feeding and growth, and easy management under laboratory conditions; (II) direct impact on agriculture by increasing soil quality and nutrients; (III) indirect impact in biocontrol of pest insects. The most well-known example of microbial-mediated mutualism in ants is that which is established between ants and aphids (Rhynchota, Aphidiae). Ants establish a long-term mutualistic interaction with aphids, managing the aphid nest and protecting them from natural aphid predators and parasites. In turn, by feeding on plant sap, aphids produce honeydew for the ants [62].

**Figure 2:**
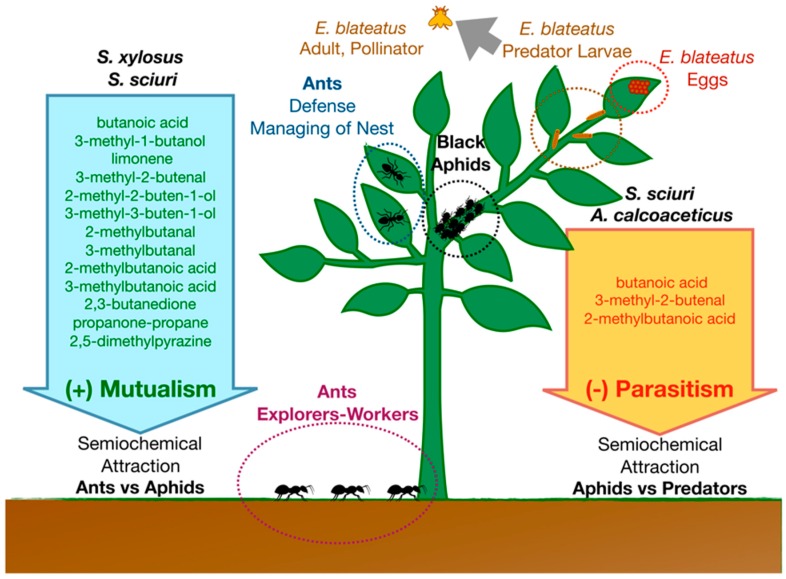
Aphids mutualism and predation. In aphids, *S. xylosus* and *S. sciuri* produces BVOCs that acts as an attractant for ants. Mutualistic ants manage and protect aphid nests. On the other hand, *S. sciuri* and *A. calcoaceticus* produce some BVOCs that help *E. blateatus* larvae to find the aphids (the prey).

In a well-characterized ant–aphid interaction model, BVOCs produced by *Acyrthosiphon pisum* (Rhynchota, Aphidiae)-associated *Staphylococcus sciuri* and *Staphylococcus xylosus* living in the honeydew produced by aphid mediate mutualistic interaction with *Lasius niger* (Hymenoptera, Formicidae) [29,30]. Bacteria produce a blend of semiochemicals (including limonene, butanoic acid, 3-methyl-1-butanol, 3-methyl-2-butenal, 2-methyl-2-buten-1-ol, 3-methyl-3-buten-1-ol, 2-methylbutanal, 3-methylbutanal, 2-methylbutanoic acid, 3-methylbutanoic acid, 2,3-butanedione, propanone-propane, 2,5-dimethylpyrazine) that attract ant scouts (Figure 2). However, at the same time, some semiochemicals produced in this system by *S. sciuri* and *Acinetobacter calcoaceticus* (butanoic acid, 3-methyl-2-butenal, 2-methylbutanoic acid) act as attractants and ovipositional stimulants for the natural enemy of *A. pisum*, *Episyrphus balteatus* (Diptera, Syrphidae). This is one of the most efficient, abundant, and highly specialized aphidophagous predators [63].

Another type of insect–insect mutualistic interaction is that between ants and butterflies during their larval stages. Interactions can be facultative or obligate, mutualistic or parasitic. In the interaction between *Myrmica* (Hymenoptera, Formicidae) and *Maculinea* (=*Phengaris*) (Lepidoptera, Lycaenidae), ants defend instars from natural enemies (usually staying on top of instars). In turn, they are rewarded with nutrition secretions from specialized exocrine glands [64]. On the other hand, this relationship can be parasitic. Caterpillars of some *Maculinea* species are adopted by certain *Myrmica* species, transported into their nest, and fed on either ant regurgitations (trophallaxis) or directly on ant brood [65]. The central role of CHCs and acoustic signals in the crosstalk between butterflies and ants is well-known [66]. The possibility that symbiotic microorganisms living on butterflies and ants may influence this host–parasite interaction by either CHC metabolism or MVOC production has recently brought the study of the butterfly and ant microbiota to attention [67,68].

**Figure 3:**
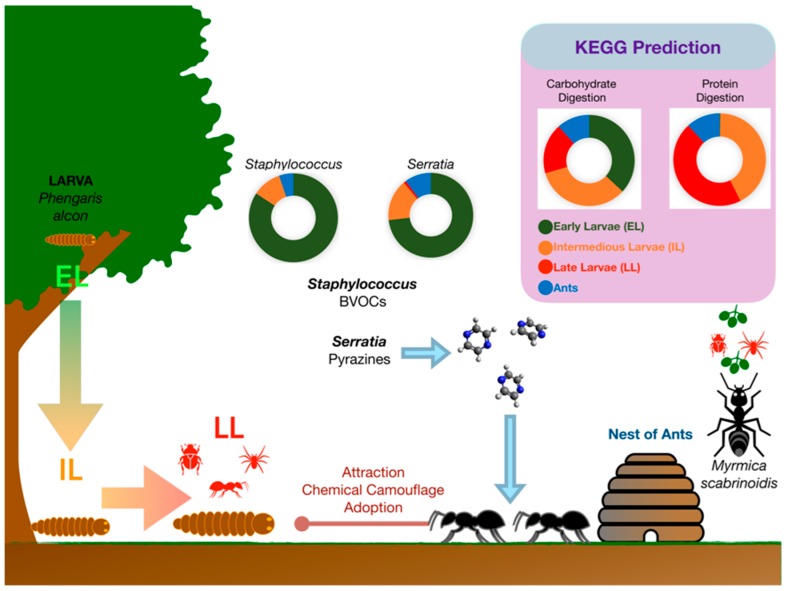
Mirmecophilus butterfly. *P. alcon (Maculinea)* is a lepidoptera that is adopted by ants (*M. scabrinodis*) in, intermediate larvae (IL) stage when the larvae fall on soil (after the early phytophagous phase). The attraction mechanism and chemical camouflage strategy is related to gut microbiota, which produces BVOCs. The genera *Staphylococcus* and *Serratia* shows a consistent distribution with camouflage skills. PICRUST prediction of genetic content shows as digestion of carbohydrate and proteins are consistent with the development of larvae and validate the system.

The gut microbiomes of the endangered butterfly *Maculinea alcon* at different life stages and that of its adopting ant *Myrmica scabrinodis* were characterized in a recent study case [67]. On the Alpine Arc, the life cycle of *Maculinea alcon* begins in August when eggs are deposited on both flower buds and leaves of *Gentiana asclepiadea* or *Gentiana pneumonanthe*. Early larves (EL) hatches through the base of the egg into the flower ovary, where they spend two to three weeks eating the flower tissue and developing seed. Phytophagous EL moults three times. Then, they chew a hole in the flower, through which they let themselves fall into the soil. Here, intermediate larvae (IL) wait until they are adopted by the ants that collect the IL and carry them to their nest. Late larvae (LL) are fed by worker ants and become carnivorous. They spend the whole autumn, winter, and spring in the ant nest. Then, in the early summer, they turn into pupae. A month later, adult butterflies emerge from pupae, which leave the ant nest and fly to flowers in July for a one-month duration [69] (Figure 3).

As demonstrated by 16S rRNA-guided metabarcoding, the *M. alcon* larval development is associated with significant changes in the structure and predicted functions of the butterfly microbiota [67]. Alphaproteobacteria dominated *M. alcon* microbiota during EL and IL stages, while gamma-proteobacteria were predominant in LL. The PICRUSt functional prediction from DNA metabarcoding data suggested changes in predominant biochemical pathways during larval development, which are consistent with a different ability to digest carbohydrates (herbivorous EL) or proteins (carnivorous LL) and to metabolize CHCs (Figure 3). A characteristic pattern was represented by an increase, among the intracellular obligate bacterial endosymbionts, in *Rickettsiella* (Gammaproteobacteria) abundance during larval development and a parallel decrease in *Wolbachia* (Alphaproteobacteria) abundance. This shift may be associated with larval color shift from red (EL) to green/yellow (LL). Indeed, *Rickettsiella* is known to be responsible for a red to green color shift in pea aphids, which makes the aphids less visible to predators and parasites [70]. A similar red to green/yellow color shift may facilitate the integration of *M. alcon* larvae in the host ant colonies considering that ant species have a dichromatic color vision system that is insensitive to red light [71].

Another characteristic pattern was represented by dynamics of BVOC-producing *Serratia* spp., *Staphylococcus* spp., and actinomycetes in *M. alcon* larvae. In particular, *Serratia marcescens* and *Serratia entomophila* were largely abundant in EL, while their abundance progressively declined in IL and LL. Microorganisms belonging to the genus *Serratia* are very common in plants, where they act as plant growth-promoting bacteria, as well as in the gut of arthropods as symbiotic bacteria. Intriguingly, these bacteria produce volatile pyrazines used as trail pheromones by insects. Particularly, 2,5-dimethylpyrazine and 3-ethyl-2,5-dimethylpyrazine are trailmarkers in ants. Thus, the abundance of *S. marcescens* and *S. entomophila* in EL and IL may play a specific role in the production of deceptive pyrazines that facilitate recognition of the *M. alcon* larvae by adopting ants.

### 2.5. Bacteria and Insect–Plant Communication

As exemplified above with the *Chrysolina herbacea—Mentha aquatica* model, bacteria play a pivotal role in plant–insect interaction systems. The vetiver grass (*Vetiveria zizanoides* (L.) Nash, syn. *Chrysopogon zizanoides* (L.) Roberty) provide us with another intriguing example. Vetiver is the only grass cultivated worldwide for to its multiple properties. The vetiver grass is very suitable for soil erosion management [72], sustaining agricultural productivity [73], and phytoremediation [74,75]. From the long roots of vetiver, an essential oil is extracted, which is used to formulate many perfumes. In addition, vetiver is extremely resistant to (or sometimes repellent to) many insect pests, although it may be often infested by pest of other crops, such as stem borers, white grubs, cicadas, and termites, which use vetiver as a refuge [76,77,78]. A study focused on *Chilo partellus*, a lepidopterous stem borer of grasses that may infest the vetiver and is a serious pest to maize, sorghum, rice, and other crops, showed that the vetiver grass is highly preferred for oviposition with respect to the aforementioned crops. However, larval survival on vetiver was extremely low, raising the possibility that this plant can be used as a plant trap around crops on which stem borers are a problem [79,80]. There is evidence suggesting that many insects use vetiver grass as refuge, and that the insect biodiversity of vetiver is actually very high including not only potential insect pests, but also a large number of general predators and parasitoids of insects [81]. The ability of the vetiver grass to attract (and manage life cycle) or repel numerous species of insects relies on the high plant potential for biosynthesis of secondary metabolites. The biosynthesis of these compounds is particularly intense in the vetiver roots that are traditionally used to repel cloth moths, head lice, and bed bugs. The vetiver oil that is extracted from the vetiver roots is a complex mixture of hundreds of sesquiterpene alcohols and hydrocarbon compounds. Several of which possess insect repellent properties. α-vetivone, β-vetivone, khusimone, zizanal, epizizanal, and (C)-(1S, 10R)-1,10-dimethylbicyclo (4,4,0)-dec-6-en- 3-one) were reported to be repellent to different insects. In particular, zizanal and epizizanal have topical irritant activity on cockroaches and flies [82]. Nootkatone, zizanol, and bicyclovetivenol are strong repellents and toxicants to Formosan subterranean termites *Coptotermes formosanus* (Isoptera, Rhinitermitidae) [83]. Indeed, the efficacy of vetiver oil and nootkatone as soil barriers against these termites was proven [84].

Intriguingly, there is evidence that root-associated bacteria may be involved in the biosynthesis of several constituents of the vetiver oil. These bacteria, living in the essential oil-producing cells and in the lysigen lacunae in close association with the essential oil, metabolize biosynthetic precursors that are synthetized by the plant [85,86]. This is supported by evidence that axenic vetiver produced only trace amounts of oil in vitro, with a strikingly different composition compared to the oils from in vivo vetiver plants. Most root-isolated bacteria were grown using oil sesquiterpenes as a carbon source and were biotrasformed into a large number of compounds typically found in commercial vetiver oils [85] (Table S1). Some of these compounds have repellent activity to insects, such as bicyclovetivenol, which was synthesized in vitro by vetiver root-associated *Pseudomonas* sp. VET-3 and *Pseudomonas* sp. VET-5 when fed on plant precursor β-caryophyllene, one of the few compounds produced by axenic vetiver [86]. This finding is consistent with a leading role of vetiver root-associated bacteria in overseeing the complex interaction between the plant and its hosted insects. Other volatile terpenoids produced by vetiver root-associated bacteria are listed in Table S1. 

Plant–insect mutualism, based on pollination performed by Hymenoptera, is established thanks to the microbiota of nectar. Rering and colleagues [87] demonstrated that yeasts (*Metschnikowia reukaufii* and *Aureobasidium pullulans*) and bacteria (*Neokomagataea* sp. and *Asaia astilbes*) produce volatiles compound in nectar. All microbial strains produced n-hexanol, while only bacteria produced the characteristic compound 2,5-dimethylfuran, while alcohols, esters, and ketones were more abundant among fungal metabolites [87]. In plant Hymenoptera systems, bacterial symbionts are also involved in seed preservation. The first insect-produced herbicide myrmicacin, which was isolated from the ants, stopped seed germination and preserved the seeds from structural damages [88]. Myrmicacin analogs play similar roles. These compounds can also stop pollen germination and mitotic division in plants and fungal cells and may inhibit fungal growth on seed surfaces [89]. Some Lactobacillales, such as *Lactobacillus plantarum,* produce myrmicacin-related hydroxy acids [90]. All these functions can be counteracted by the production of antibiotics by Actinomycetales in the “fungal garden”, a specialized structure managed by ants that cultivate fungi as a source of food [91].

Another interesting point is the ability of nonpathogenic bacteria to mediate physiological response and signaling pathways in plants. A review by Dicke shows that phytobiome, which is formed by microbiome and macrobiome (insect), manages phenotypic plasticity in plants, inducing changes in VOCs emitted by the plant and influencing the relationship between herbivores and plants [92]. Some nonpathogenic bacteria cause transcriptomic and metabolic changes in plants. For example, root-colonizing *Pseudomonas fluorescens* SS10 induces resistance against another pathogenic *Pseudomonas* species (*P. syringae* pv *tomato*, *Pst*), but also enhances resistance against the insect pest *Spodoptera exigua* (Lepidoptera, Noctuidae). This induction of resistance depends on a signaling pathway based on salicylic acid, while other signaling pathway systems are based on phytohormones as jasmonic acid and ethylene. Using *Arabidopsis thaliana*, Van de Mortel and coworkers identified approximately 50 metabolites related to the salicylic acid signaling pathway and demonstrated that camalexin and glucosinolate were principal compounds related to resistance against *Pst*, using gene-disruption approach to silence signaling [93].

Another study showed that the root colonization by *P. fluorescens* in *A. thaliana* influences not only the defense plant response to herbivores, but also the relationship between herbivores and their parasitoid. As shown in experiments with *A. thaliana*, the VOCs profile emitted by plants infested by aphid *Myzus persicae* (Hemiptera, Aphididae) is less attractive for the parasitoid *Diaeretiella rapae* (Hymenoptera, Braconidae) if the roots of the plant are colonized by root bacteria [94]. This phenomenon is lost when the jasmonic acid production is silenced in the plant [94]. The jasmonic acid signaling pathway is related also to the mediation of plant defenses in bitrophic systems. Caterpillars of *Pieris brassicae* (Lepidoptera, Pieridae) develop faster and bigger when the plants are simultaneously infected by aphid *Brevicoryne brassicae* (Hemiptera, Aphididae). This phenomenon can occur because the infestation of aphids reduces the levels of jasmonic acids by ten-fold as a consequence of low transcriptions of the *lox* and *myc* genes, coding for enzyme-related to jasmonic acid biosynthesis [95]. The facilitation also affects the way herbivores influence parassitoids. As reported in the example described previously, the development of the parasitic wasps *Cotesia glomerata* (Hymenoptera, Braconidae) is faster if the parasitized caterpillars are fed on by plant–infested aphids, probably because a better fitness of herbivorouse hosts leads to a better fitness of parasitoid [95].

The third trophic level, represented by parasitoid of herbivores, appears to be most influenced by symbionts. *C. glomerata* injects in the egg of herbivores, such as *P. brassicae* and *P. xylostella* (Lepidoptera, Plutellidae), venom, and the symbiotic polydnaviruses (PDVs) that sustain the development of parasitoid. This interaction caused an altered plant-mediated interaction that reduced colonization of other herbivores [96]. On the other hand, symbiotic interaction can protect the pray to parasitoid as shown for aphids, attenuating herbivore-induced plant volatiles [97,98].

## 3. BVOC Biosynthetic Pathways and Their Intersections with Primary and Secondary Metabolism

The volatile molecules produced by bacteria play a primary role in the chemical communication between insects. They share common features including low molecular mass, high vapor pressure, low boiling point, and low polarity, although they may be produced through very different biosynthetic pathways. The aim of this section was to connect entomology to industrial microbiology using a biochemical point of view, implementing a classification method for semiochemical compounds based on microbial source and pathway of biosynthesis (Table S1). The mechanisms of biosynthesis are also interesting for an industrial purpose: The fermentation of biomass to produces semiochemical useful to manage pests and insects. In this section, we attempted to illustrate some validated or proposed pathways underlying the production of these compounds. The output was an atlas (Table S1) that links each BVOC that acts as a signaling molecule in insects to the respective producing microorganism(s) and biosynthetic pathway(s). For this purpose, the atlas was hyperlinked to both KEGG and Pherobase databases. Excel’s formatting allowed a suitable sorting of data according to consultation needs. Some biosynthetic pathways led to the production of BVOCs through primary and secondary metabolism. These pathways are illustrated below.

### 3.1. BVOCs and Primary Metabolism

A number of BOVCs may be produced as intermediates, byproducts, or endproducts of the bacterial primary metabolism through well-conserved pathways. Most of these pathways are fundamental for the energy metabolism, for the biosynthesis of cellular structure, and for bacterial growth and survival. Simple carboxylic acids, such as formic acid, acetic acid, lactic acid, and succinic acid, may be produced by mixed acid fermentation in facultative anaerobic enteric bacteria. When their intracellular concentration is high as a result of an intense metabolic activity, the molecules are released passively in the environment as BVOCs. Other enteric bacteria may vigorously produce acetoin (3-hydroxybutan-2-one) and its reduced form 2,3-butanediol through butanediol fermentation. Several clostridia possess the acetone-butanol-ethanol fermentation that produces propanone and butanol. Clostridia are strictly anaerobic bacteria [99] that, thanks to this peculiar energy metabolism pathway, are used to produce solvent or fuels including hydrogen [100]. *Propionibacterium-* and propionate-fermenting microorganisms are other bacteria with a peculiar energy metabolism that contributes to propionate production from succinate. Many fermentative pathways lead to acetate production in addition to other metabolic processes such as those occurring in strictly anaerobic homoacetogenic bacteria.

Amino acid metabolism by the Ehrlich pathway [101] through the catabolism of branched-chain amino acids (leucine, valine, and isoleucine), aromatic amino acids (phenylalanine, tyrosine, and trytophan), and the sulfur-containing amino acid (methionine) leads to the formation of fusel acids and fusel alcohols, and many of these BVOCs may act as semiochemicals. In particular, 2-phenylethanol and phenylacetic acid are produced by phenylalanine catabolism. Additionally, guaiacol and phenol are formed through tyrosine catabolism, while the indole is a direct precursor of tryptophan. Many BVOCs can be produced through the catabolism of branched-chain amino acids. It is interesting to note that research in this field intersects with biotechnology. A number of compounds, which act as chemical signals, are also studied for some biotechnological applications. An example is biofuel production using *Corynebacterium glutamicum* that was engineered for producing 2-methyl-1-butanol and 3-methyl-1-butanol via the Ehrlich pathway from 2-keto-3-methylvalerate and 2-ketoisocaproate, respectively [102]. Another example is provided by an *E. coli* strain that was genetically engineered to implement the production of 2-methyl-1-butanol, 3-methyl-1-butanol and 2-phenylethanol using 2-keto-isocaproate 2-keto-methyl-valerate 2-keto-isovalerate 2-phenyl-pyruvate as a substrate [103,104].

Volatile sulfur compounds are produced through methionine catabolism. Methionine gamma-lyase is central in this catabolic pathway. This enzyme directly degrades sulfur-containing amino acids to α-cheto acids, ammonia, and thiols [105]. Methionine is decomposed by various bacteria with production of methanethiol, dimethyl sulfide, and dimethyl disulfide [106,107]. All these compounds, particularly dimethyl disulfide, have proven biological activities against insects, and their use in insect pest management has been proposed as repellent [108].

Other BVOC-producing pathways are linked to biological matter processing, including the catabolism of fatty acids and lipid degradation, aromatic compound degradation, and protein and carbohydrate degradation. Catabolism of diet-introduced macromolecules by gut bacteria have a central role in the biosynthesis of semiochemicals such as those that are produced by the aromatic compound degradation pathways, i.e., benzaldehyde, benzoic acid, benzyl alcohol, guaiacol (2-methoxyphenol), phenol, phenylacetic acid, phenylethanol, phenylmethanol, and propionate. Many of these pathways have some intersections between them and, therefore, common intermediates. For example, propionate metabolism involves propionate and propanone, which is also relevant in the metabolism of ketone bodies, while aminobenzoate degradation involves guaiacol.

### 3.2. BVOCs and Secondary Metabolism

A number of specialized BVOC-producing pathways are linked to secondary metabolism. Bacterial secondary metabolism includes biochemical processes that, basically, are not essential for bacterial life, but play key roles in bacterial adaptation to unfavorable conditions, environmental response, and ecosystem regulation at the biodiversity level through species competition [109]. These functions are mostly encoded by genes outside the microbial “core genome” and are subject to intraspecies variability, although precursors of secondary metabolites come from primary metabolic pathways. Here, well-characterized or proposed pathways leading to three major classes of BVOCs that act as chemical signals in insects are illustrated. These classes include (I) pyrazines, (II) terpenes and terpenoids, and (III) alkanes and alkenes.

#### 3.2.1. Pyrazine Metabolism in Bacteria

Pyrazines, an important class of BVOCs with chemical signaling properties in insects, are secondary metabolites that have been known since the early 1970s for their use in agriculture (insecticides and nematicides) or medicine (antidepressants, diuretics, and antibacterial) [110]. More recently, these compounds were described as flavor ingredients in the industries of chocolate and coffee [111], and also as anti-neoplastic [112] and anti-HIV drugs [113]. The pyrazine biosynthetic pathways exemplify how primary and secondary metabolisms are strictly interconnected. In general, in a secondary metabolism biosynthetic pathway, the first enzyme of a secondary biosynthetic pathway controls the metabolic flux, channeling precursors from the primary metabolites (amino acids, in pyrazine biosynthesis) toward specialized secondary metabolites. Thereafter, different sets of reactions occur for precursors assembling in the molecular skeleton and post-assembly modification.

Production of pyrazines for commercial purposes have allowed a better understanding of both the physiology of the pyrazine-producing microorganisms and the details of the biosynthetic pathways underlying their synthesis. Although there are many studies that have treated the biosynthesis and production of bacterial pyrazines as secondary metabolites, only few studies have reported their possible activity as semiochemicals in insects. We know, for instance, that 2,5-dimethylpyrazine is an important pheromone in ants *Atta sexdens* and it is produced by *Citrobacter freundii* [114] and *Microbacterium foliorum* [115]. In addition, 2,5-dimethyl-pyrazine and other pyrazines (2,3,5-trimethyl-pyrazine and tetramethyl-pyrazine) are produced by bacteria of the genus *Bacillus* by industrial fermentations [116]). Rajini and coworkers [117] reviewed some industrial applications of microbial pyrazines, the producer strains, and the proposed biosynthetic pathways.

**Figure 4:**
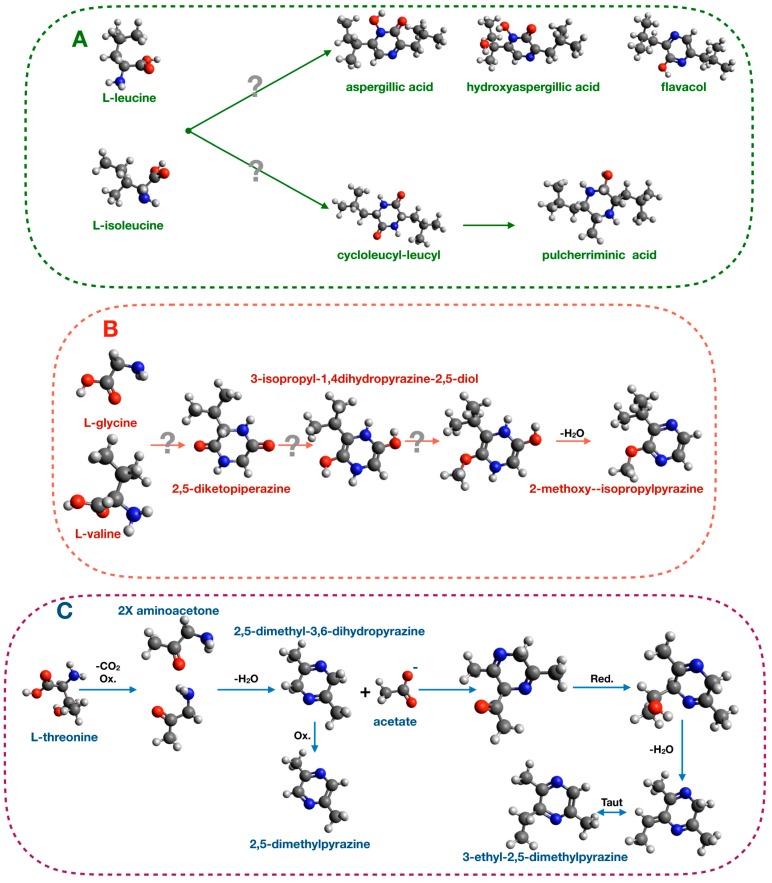
Pyrazines biosynthesis. Generally, experimental data show a correlation between amino acids metabolisms and pyrazine production in microorganisms. Yeast species, if cultured in isoleucine or leucine-rich medium, produce pyrazines used in an industrial process (**A**) *Aspergillus* produces aspergillic acid and similar molecules, *Candida pulcherrima* produces pulcherriminc acid. A similar effect (**B**) is observed in *Pseudomonas perolens*, adding valine and glycine in the culture broth. In this last case, there was a proposed pathway. Finally, adding radiolabeled precursors, a pathway was individuated in *Serratia marcescens* B2 that start from threonine (**C**).

Many studies demonstrated that pyrazine biosynthesis is tightly linked to amino acid metabolism. For examples, 2,5-diisopropyl-pyrazine, 2-isopropyl-5-secbutyl-pyrazineand, and 17 other pyrazines were produced upon valine feeding of *Paenibacillus polymyxa* cultures [118]. These BVOCs are also produced by bacteria of the genus *Exiguobacterium* and act as oviposition signals in *Anopheles gambiae* [32,33]. In yeasts of the genus *Aspergillus* (*Aspergillus flavus* and *Aspergillus oryzae*), a similar experiment of amino acid feeding demonstrated that leucine and isoleucine supplementation of the culture medium enhanced the production of aspergillic acid [119]. This compound has antibacterial and chemoprotective activities [120,121]. Other pyrazines produced by the yeast of the genus *Aspergillus* (*Aspergillus sclerotorium* [122] and *Aspergillus parasiticus* [123]) are listed in Table S1. The proposed biosynthetic mechanism is based on condensation reaction between leucine and isoleucine and is depicted in Figure 4.

Another pyrazine, pulcherriminic acid, (2,5-diisobutyl-3,6-dihydroxypyrazine-1,4-dioxide) is synthesized by various bacteria, including *Bacillus subtilis*, *Micrococcus violagabriellae,* and *Paenibacillus maceran*, as well as by the yeast *Candida pulcherrima* [119,120,121,122,123,124,125,126,127]. In *B. subtilis* and *C. pulcherrima,* the proposed pathway for biosynthesis of this pyrazine produces a cyclic dipeptide cyclo l-leucyl-l-leucyl intermediate that is then converted in pulcherriminic acid [127]. Another proposed pathway in *Pseudomonas perolens* starts from valine and glycine to generate 2-methoxy-3-isopropylpyrazine [128] (Figure 4).

Several bacteria belonging to the Enterobacteriaceae family, particularly bacteria of the *Serratia* genus, are well-known for their ability to produce pyrazines. Specifically, *Serratia oderifera*, *Serratia ficaria*, and *Serratia rubidea* synthesize the alkylated pyrazines 3-isopropyl-2-methoxy-5-methylpyrazine, 3-isopropyl-2-methoxy-methylpyrazine, and 3-isobutyl-methoxy-pyrazine) [129]. A recent study demonstrated that L-trenonine and acetic acid are the precursors for 2,5-dimethylpyrazine and 3-ethyl-2,5-dimethylpyrazine synthesis in *Serratia marcescens* [37]. Radiolabeled precursors were used in this study and their incorporation into the final compounds was shown, leading to the proposed pathway illustrated in Figure 4.

Other microorganisms contributing to chemical signaling in insects as biotransforming agents include plant-derived pyrazines introduced by diet, which metabolized by some bacterial strains using two major metabolic pathways [117]. The first one converts hydroxy-pyrazines to glycine by oxidative degradation as reported in bacteria of the genus *Pseudomonas* [130], while the other one, which was reported in bacteria of the genus *Stenotrophomonas*, metabolizes hydroxy-pyrazines through an initial reduction, although the pathway is not well-characterized [131]. Another interesting example of pyrazine catabolism occurs in *Mycobacterium tuberculosis*. In vivo, this microorganism is sensitive to pyrazinamide, a first-line drug recommended by the World Health Organization for the treatment of tuberculosis in 1995. The drug, in vivo but not in vitro, is converted to pyrazinoic acid by *pncA*-encoded Mn^2+^/Fe^2+^ nicotinamidase [132]. Pyrazinoic acid inhibits FAS I (Fatty acids synthase I) and acidifies the microenvironment [133], causing the subsequent disruption of the membrane potential and disassembly of the membrane [134].

#### 3.2.2. Terpene and Terpenoid Metabolism in Bacteria

Terpenes, terpenoids, and, more in general, isoprenoids, are a large class of compounds widespread among plants, yeasts, archaea, and bacteria with various functions, such as hormones [135], photosynthetic pigment [136], and major structural components in the archaeal membrane [137]. The isoprenoid class includes cholesterol and liposoluble vitamins A, D, E, and K [138]. In plants, many terpenes and terpenoids are multifunctional compounds. They protect plants against herbivores and pathogens, attract mutualists such as pollinators, and may act also as chemical signals for other plants or insects [139,140].

Different species of *Dendroctonus* are parasites of conifers, which are plants that produce various terpenes useful for protecting themselves from herbivorous insects [141]. The insect gut community affects tolerance to terpenes and terpenoids. Some bacterial strains metabolize these dangerous compounds, thereby reducing their toxicity and improving the insect survival [142]. On the other hand, in *Dendroctonus valens* multifunctional pheromone verbenone is synthesized by the gut bacterial community by biotransforming conifer monoterpenes ingested by diet [23,24], showing the promiscuous nature of these compounds.

Other terpenes act as hormones in plants, such as in the case of gibberellins, a class of diterpenes. In plants, gibberellins control development at different levels including seed germination, stem elongation, and flower induction. Plants, bacteria, and fungi synthesize these compounds, but the biosynthetic pathways are distinct in the different organisms. Plants use two diterpene synthases to form *ent*-kaurene, the giberellin precursor, from geranylgeranyl diphosphate (GGPP), while fungi use only a single bifunctional diterpene synthase. In both plants and fungi, *ent*-kaurene is then oxidized to *ent*-kaurenoic acid by cytochromes P450. *ent*-kaurenoic acid is further metabolized by different sets of enzymes in plants (sequentially: Two cytochromes P450, two 2-oxoglutarate dependent dioxygenases) and fungi (sequentially: Two cytochromes P450, desaturase, cytochrome P450) to obtain the final product, gibberellin A (GA_3_) [143]. Bacteria have evolved a distinct pathway, as shown in *Bradyrhizobium japonicum*, which contains an operon consisting of genes coding for a ferredoxin, a short-chain alcohol dehydrogenase, three cytochrome P450, a GGPP synthase, and two diterpene synthases (*ent*-copalyl diphosphate synthase and *ent*-kaurene synthase), which control giberellin biosynthesis.

To synthesize terpenes and terpenoids, two interconvertible building blocks are required: Isopentenyl pyrophosphate (IPP) and dimethylallyl pyrophosphate (DMAPP). Biosynthesis of these precursors involves two pathways, i.e., the mevalonate pathway (MVA) and the non-mevalonate pathway, also known as 2-*C*-methyl-d-erythrol 4-phosphate (MEP) pathway [144,145]. The MVA pathway is considered as the fundamental pathway for isoprenoid biosynthesis in archaea and eukaryotes, although it seems to be present also in several bacteria. There are three hypotheses about the evolution of MVA pathway [145]. The first hypothesis contends that an ancestral pathway appeared in a common ancestor of eukaryotes and archaea, and it was recently acquired by few bacteria by horizontal genetic transfer (HGT) from a eukaryotic or archaeal donor. The second one proposes a similar model in which the HGT occurred in a common ancestor of a group of modern-day bacteria. The third hypothesis proposes that the MVA pathway was ancestral not only to archaea and eukaryotes, but also to bacteria. However, it was lost by most of bacteria. In this scenario, the last universal common ancestor life (LUCA) possessed the MVA pathway [145]. A number of Actinobacteria, Bacterioidetes, Firmicutes, Chloroflexi, Proteobacteria, and Spirochetes genomes contain homologs of MVA pathway genes, but not the entire classic pathway [145]. It has been proposed that the non-individuated MVA pathway enzymes likely evolve independently to complete the pathway, are supplementary enzymes that can be provided in *trans* configuration by other pathways, or are moonlighting proteins with two or more functions [146,147].

**Figure 5:**
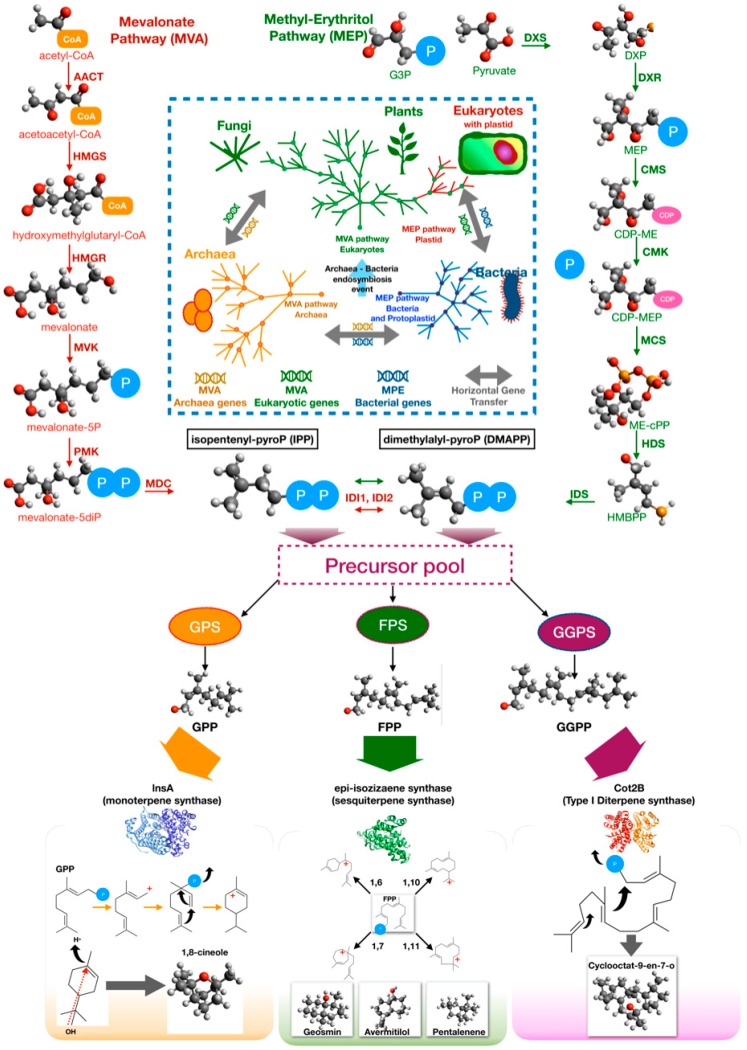
Terpene and terpenoids biosynthesis. The precursors for biosynthesis of terpenes and terpenoids (isopentyl-pyroP, IPP and dimethylallyl-piroP, DMAPP) are synthetized by two biosynthetic pathways: The mevalonate pathway (MVA, in red) and the methylerythritol phosphate pathway (MEP, in green). As shown in the the square with blue dotted line, MVA was found in eukaryotes and archaea, while MEP was found in bacteria or in eukaryotes equipped with plastid. Horizontal gene exchange has allowed the acquisition of MVA (for the bacteria) or MEP genes (for the eukaryotes). GPS (geranyl-pyroP synthase), FPS (farnesyl-pyroP synthase) and GGPS (geranylgeranyl-pyroP synthase) assemble IPP and DMAPP into linear long-chain precursors used by the biosynthetic enzymes to produce the terpenes-like ring. In the figure, the examples are reported (from precursor to ring cyclization), including monoterpene synthases, diterpene synthases, and sesquiterpene synthases. Abbreviations: MVA: AACT = acetoacetyl-CoA thiolase, HMGS = 3-hydroxy-3-methylglutaryl- CoA synthase, HMGR = 3-hydroxy-3-methylglutaryl- CoA reductase, MVK = mevalonate kinase, PMK = phosphomevalonate kinase, MDC = mevalonate- 5-decarboxylase. Abbreviation of MEP: DXS = 1-deoxy-d-xylulose-5-phosphate synthase, DXP = deoxy-d-xylulose-5-phosphate, DXR = 1-deoxy-d-xylulose-5-reductase; MEP = methyl-erythritol phosphate, CMS = MEP cytidylyltransferase, CDP-MEP = cytidylyl-MEP, MCS = ME-cPP synthase, ME-cPP = methyl-erythritol 2,4-cyclodiphosphate (ME-cPP), HDS = hydroxymethylbutenyl 4-diphosphate synthase, HMBPP = hydroxymethylbutenyl 4-diphosphate, IDS = IPP/DMAPP synthase, IDI1/IDI2 = IPP-DAMPP isomerase.

Details of the MVA pathway are illustrated in Figure 5. The first three steps, called the lower mevalonate pathway, are catalyzed, respectively, by acetoacetyl-CoA thiolase (AACT), HMG-CoA synthase (HMGS), and HMG-CoA reductase (HMGR) [148]. The result of lower pathway is the key precursor mevalonate and is then converted to mevalonate 5-phosphate by mevalonate kinase (MVK). This molecule is converted into IPP by two reactions which, in eukaryotes, involve phospho-mevalonate kinase (PMK) and mevalonate-diphosphate decarboxylase (MDC) while, in archaea, they involve a putative phospho-mevalonate decarboxylase (MPD) and a characterized isopentenyl phosphate kinase (IPK) [148]. Isomerization of IPP to DMAPP is carried out by distinct isopentenyl pyrophosphate isomerases in eukaryotes (IDI1) and archaea (IDI2) [148].

There is evidence that the MVA pathway exists and is essential in several bacteria, including staphylococci, streptococci, and enterococci, at variance with *Bacillus subtilis* and gram-negative bacteria that possess the MEP pathway. For example, in *S. aureus,* the mevalonate kinase was isolated and well-characterized [149]. Furthermore, knockout of the MVA pathway genes of *Streptococcus pneumoniae* stopped the growth in vitro under standard conditions. The growth was restored by adding mevalonate to the culture medium, demonstrating that this pathway is essential in this pathogenic bacterium and might represent a target for new antimicrobial compound [150].

The MEP is diffused in bacteria and plants. MVA and MEP coexist in eukaryotic organisms equipped with a plastid that evolved from a photosynthetic microorganism similar to modern-day cyanobacteria, according to the endosymbiotic theory [151]. These pieces of evidence suggest that MEP is characteristic of prokaryotes, and it was later acquired by eukaryotes with the photosynthetic endosymbiont, although different origins have been proposed [152].

Details of the MEP pathway are illustrated in Figure 5. The MEP pathway begins with the addition of pyruvate to glyceraldehyde-3-phosphate with the decarboxylation of pyruvate to yield 1-deoxy-d-xylulose-5-phosphate (DXP) and CO_2_. This reaction is catalyzed by DXP synthase (DXS) [153]. However, the same reaction occurs in E1 subunit of pyruvate kinase or decarboxylase as moonlighting activity [154,155]. The next steps are wel- characterized and are catalyzed by enzymes encoded by 6 *isp* genes: *ispC*, *ispD (ygbP)*, *ispE (ychB)* and *ispF (ygbB)*, *ispG (gcpE)*, *ispH (lytB)* [153]. DXP is reduced to 2-*C*-methyl-d-erythritol 4-phosphate (MEP) by DXP reductoisomerase (IspC, DXR) [156,157]. MEP is converted 4-diphosphocytidyl-2-*C*-methyl-d-erythritol (CDP-ME) by CDP-ME synthase (IspD, CMS) using CTP [158]. CDP-ME kinase (IspE, CMK) phosphorylates CDP-ME yielding 4-diphosphocytidyl-2-*C*-methyl-d-erythritol 2-phosphate (CDP-MEP); 2-*C*-methyl-d-erythritol 2,4-cyclodiphosphate synthase (IspF, MCS), then detaches the CMP and converts the intermediate into phosphocyclic compound 2-*C*-methyl-d-erythritol 2,4-cyclodiphosphate (ME-cPP) [159,160]. Finally, hydroxy-methyl-butenyl 4-diphosphate [HMBPP] synthase (IspG, HDS) catalyzes the synthesis of HMBPP from ME-cPP, while 4-hydroxy-3-methylbut-2-enyl-diphosphate reductase (IspH, HDR) synthesizes a mixture of IPP and DMAPP [161]. In the MEP pathway, as well as in the MVA pathway, an isomerase (IDI) catalyzes the interconversion of IPP and DMAPP [162].

Although all terpenes are synthesized from two common precursors, the basic C5 isoprene units IPP and DMAPP, the structural and functional diversity of the final products are surprisingly high. Such a diversity is largely dependent on the biosynthetic enzymes that process the precursors to form the terpene rings [143]. The head-to-tail condensation of IPP to DMAPP results in the formation of geranyl pyrophosphate (GPP), and the further addition of another IPP unit leads to production of farnesyl pyrophosphate (FPP). Geranylgeranyl pyrophosphate is formed by the condensation of one FPP with one IPP unit. GPP, FPP, and GGPP are, respectively, the acyclic precursors for the biosynthesis of monoterpene, sesquiterpene, and diterpene, which are the most common terpenes in bacteria (Figure 5). Cyclization reactions are catalyzed, respectively, by monoterpene cyclases, sesquiterpene cyclases, and diterpene cyclases.

The first bacterial monoterpene synthase (encoded by *cnsA*) was isolated from *Streptomyces clavuligerus* ATCC 27064 by Ohnishi and coworkers [163]. This enzyme catalyzes the biosynthesis of 1,8-cineole from GPP involving an α-terpineol intermediate until the complete conversion. The reference monoterpene synthase, 2-methylisoborneol synthase, instead uses (E)-2-methyl-GPP as a substrate. The product, 2-methylisoborneol, is volatile homoterpene alcohol with an earthy, musty odor. The enzyme mechanism is well-understood and involves ionization of the substrate followed by isomerization and final cyclization [164]. A second monoterpene synthase was identified in another *Streptomyces clavuligerus*, the linalool/nerolidol synthase (*lnsA*). This enzyme catalyzes the synthesis of the acyclic terpenoids (*3R*)-linalool and (*3R*)-(*E*)-nerolidol using, GPP and FPP as substrates, respectively [165]. Finally, as a moonlighting function, several sesquiterpene synthases may also produce monoterpenes using GPP, an unusual substrate for this type of terpene synthase. This hypothesis was demonstrated by heterologous expression in *E. coli* of the epicubebol synthase from *Streptosporangium roseum* [166].

Sesquiterpenes synthases are the major class of terpene synthases that are found in bacteria. These enzymes use FPP as substrate, but different enzymes can cyclize the precursors with different mechanisms, resulting in various ring closures: 1,6 (bisabolyl catione), 1,7 (cycloheptenyl cation), 1,10 ((E,E)-germacrenyl cation), and 1,11 ((E,E)-humulyl cation). Usually, when an ionic intermediate is involved in the biosynthetic pathways, an enantiomer mixture may result from the reaction, thereby expanding the repertoire of synthesized molecules [167]. A typical sesquiterpene synthase is pentalenene synthase from *Streptomyces exfoliates*, an enzyme related to the synthase of pentalenolactone, which was isolated from *Streptomyces roseogriseus* in 1957 as an antibiotic [168]. Geosmin synthase is another common sesquiterpene synthase in streptomycetes. Geosmin (l,2,7,7-tetramethyl-2-norborneol) is a terpene compound that is responsible for the earthy scent released when rain falls on dry soil. The gene encoding this biosynthetic activity was first identified in the model organism *Streptomyces coelicolor* A3(2) [169]. It is interesting to note that 2-methylisoborneol and geosmin, two rather common BVOCs produced by many streptomycetes, have powerful biological effects against insects. A system and method for attracting ants by geosmin and/or repelling ants by 2-methylisoborneol was patented [170].

Diterpene synthases are a complex and relatively misunderstood class of enzymes that catalyze the conversion of GGPP into diterpene molecules. Two types of diterpene synthases (type I and II) can be distinguished. Type I diterpene synthases transform directly the substrate into the final terpenes, while type II diterpene synthases catalyzes the synthesis of diphosphate intermediates that can be processed by type I cyclases. The reference enzyme for type I diterpene synthases is cyclooctat-9-en-7-ol synthase (CotB2), which was isolated from *Streptomyces melanosporofaciens* MI614-43F2. This enzyme is involved in the biosynthesis of cyclooctatin, a lysophospholipase inhibitor [171,172]. The first bacterial type II diterpene synthase reported in literature was the terpentedienyl diphosphate synthase from *Streptomyces griseolosporus* MF730-N6, which catalyzes the synthesis of terpentedienyl diphosphate that is converted into terpentetriene, the precursor of the antibiotic terpentecin [173,174].

#### 3.2.3. Alkane and Alkene Metabolism in Bacteria

CHCs play a central role in social insect communication and influence various aspects of their behavior and health. Many studies have focused on CHCs as both pheromones and signaling molecules. However, few studies have addressed their biosynthetic pathways, which therefore remain poorly characterized. Initial studies identified the biosynthetic source of CHCs in the insects’ cells only, but further studies demonstrated the relevance of the microbiota metabolism as described above. Many studies report the ability of certain groups of bacteria to synthesize linear alkanes and alkenes, but even in this case, the biosynthetic pathways remain poorly characterized, with some exceptions. Most of the information comes from studies on cyanobacteria [175].

**Figure 6:**
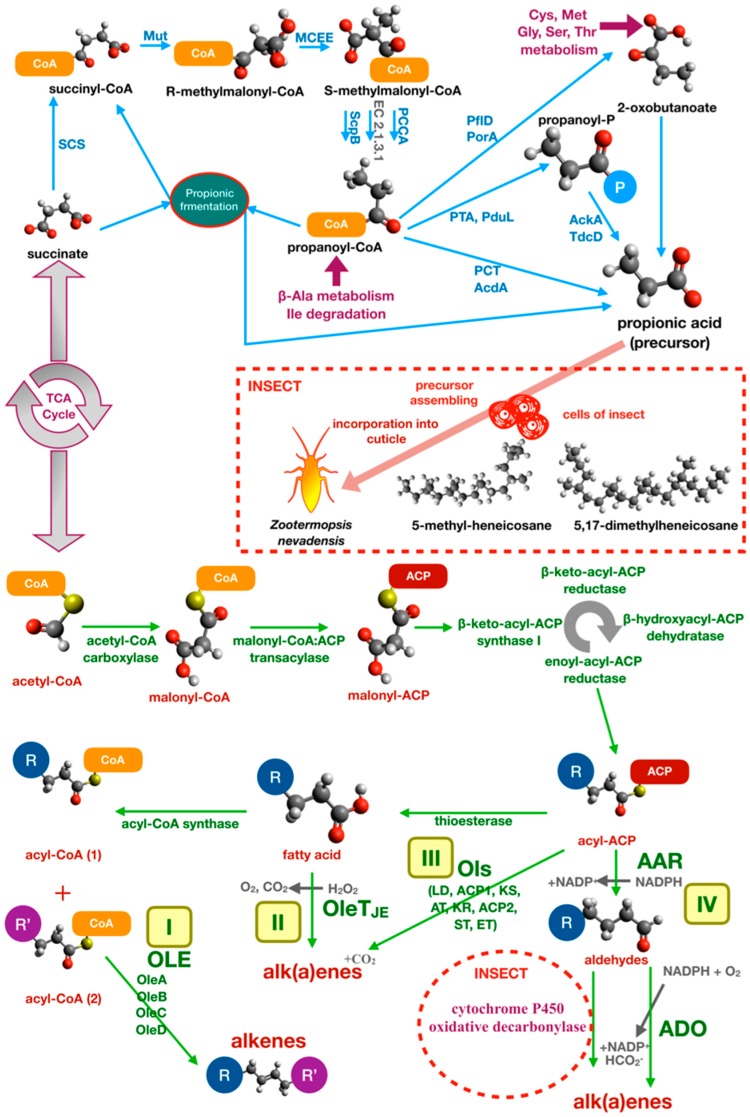
Linear hydrocarbons biosynthesis. Microorganisms produce hydrocarbons through different biosynthetic pathways. The blue arrows indicate bacterial metabolism for a precursor of hydrocarbons (propionic acid). As shown, different pathways produce propionate including fermentation, biotransformation of succinate, and biotransformation of intermediates from amino acid metabolisms. In *Z*. *nevadensis*, propionate is produced by bacterial community starting from succinate and is then assembled in final cuticular hydrocarbons by the insect. The green arrows show the direct synthesis of alkanes and alkenes in bacteria. There are four pathways: AAR/ADO, Ols, OleABCD, and OleT_JE_. Abbreviations: SCS = succinyl-CoA synthetase, MUT = methylmalonyl-CoA mutase, MCEE = methylmalonyl-CoA/ethylmalonyl-CoA epimerase, ScpB = methylmalonyl-CoA decarboxylase, PCCA = propionyl-CoA carboxylase alpha chain, EC 2.1.3.1 = methylmalonyl-CoA carboxytransferase, PflD = formate C-acetyltransferase, PorA = pyruvate ferredoxin oxidoreductase alpha subunit, Pta = phosphate acetyltransferase, PduL = phosphate propanoyltransferase, AckA = acetate kinase, TdcD = propionate kinase; Pct = propionate CoA-transferase, AcdA = acetate-CoA ligase (ADP-forming) subunit alpha.

Different biosynthetic pathways leading to alkanes and/or alkenes from fatty acid precursors were proposed: (I) the Acyl-ACP reductase-aldehyde-deformylating oxygenase pathway (AAR-ADO), (II) the olefin biosynthetic pathway (Ole, encoded by the *oleABCD* operon), (III) the terminal olefin pathway (OleT_JE_), (IV) the olefin synthase pathway (Ols) (Figure 6). Of all these pathways, only the first two (AAR-ADO and Ole) are well-characterized [175].

The AAR-ADO was first identified in *Synechococcus* [176] using a differential and subtractive genomic approach based on the differences between alkane/alkene producing and nonproducing strains. Evidence was provided that alkanes and alkenes biosynthesis start from an acyl-acylated acyl-carrier protein (ACP). The acyl-ACP substrate is then reduced by acyl-ACP reductase (AAR) and the resulting aldehyde is converted to terminal alkane or alkene by aldehyde-deformylating oxygenase (ADO) [177]. ADO is a unique enzyme of cyanobacteria, which converts acyl aldehyde precursors into alkane/alkene by the combined action of ferredoxin and NADPH:ferredoxin/flavodoxin oxidoreductase and O_2_ as an electron acceptor [177].

The Ole pathway was characterized in *Micrococcus luteus* [167]. The *M. luteus* olefin biosynthetic operon *oleABCD* is composed of three genes that codes for enzymes: Thiolase (OleA), bifunctional α/β hydrolase and AMP-dependent ligase/synthetase (OleB/C), and short-chain dehydrogenase (OleD) [178]. The pathway starts with a nondecarboxylative Claisen condensation catalyzed by OleA, which generates a β-keto acid that is then reduced by OleD in a NADPH-dependent reaction to produce a β-hydroxy acid. OleC catalyzes the conversion of the β-hydroxy acid to an alkene using ATP. OleB is thought to perform scaffolding or regulatory function in the Ole complex. This pathway generates nonterminal long-chain alkenes by fatty acid head-to-head condensation.

Terminal olefins are, instead, produced by *Jeotgalicoccus* using OleT_JE_, a fatty acid decarboxylase that was annotated as P450 peroxygenase of cyp152 family. OleT_JE_ uses H_2_O_2_ as an electron source to decarboxylate fatty acids releasing H_2_O and CO_2_ [179].

The Ols pathway was described in *Synechococcus*. In this photosynthetic cyanobacterium, the olefin synthase (CurM), a multifunctional protein very similar to polyketide synthases (PKSs), was characterized. As well as bacterial PKS involved in the synthesis of antibiotics, CurM is composed of multiple domains: A loading domain (LD), two ACP domains (ACP1 and ACP2), a central ketosynthase (KS) domain, an acyltransferase (AT) domain, a ketoreductase (KR), a sulfotransferase (ST) domain, and a carboxy-terminal thioesterase (TE) domain [180].

In addition to the example related to pyrazines, biotechnological exploitation of the ability of certain bacteria to produce alkanes/alkenes is leading to a better understanding of the underlying biosynthetic pathways. An artificial pathway (CAR/FAR-ADO) was implemented in *E. coli* to produce precursor (aldehydes) using (I) carboxylic acid reductase (CAR) from *Mycobacterium marinum*, (II) phosphopantetheinyl transferase Sfp from *Bacillus subtilis*, and (III) fatty acid reductase (FAR) complex (LuxC, LuxE, and LuxD) from *Photorhabdus luminescens*. This precursor was converted by ADO to produce hydrocarbons [181,182].

Although alkanes and alkenes can be produced by bacteria, insects can contribute to their synthesis. For example, in *Drosophila*, one enzyme (P450) of the CYP4G family oxidatively produces hydrocarbons, starting from aldehydes precursors [183].

## 4. Conclusions and Perspectives

Despite the involvement of microorganisms, the chemical communication between insects and plants is now recognized due to numerous studies. However, many questions still remain unanswered. Many of these concerns arise from the fact that the majority of studies in this research area were mostly observational. They allow us to associate certain phenotypes (or behaviors) in insects with a specific structure of the insect microbial community, but they do not allow us to detect the cause–effect relationships. On the other hand, experimental in vitro studies are often limited in the validity of the system or in the analysis model. Very few experimental studies are carried out in the natural environment.

In other words, in many cases, there is very clear observational evidence regarding the role played by several microorganisms in such transkingdom interactions. In some cases, there is also clear evidence that these microorganisms can produce in vitro molecules that may act as chemical signals in insects and/or plants. On the contrary, in most cases, formal proofs are often missing (I) that there is production of these bioactive molecules in the natural environment where these microorganisms live, and (II) that their amount is sufficient to elicit a biological response in the host. The measurement of microbial activity in the natural environment is very difficult, especially when it is necessary to quantify compounds that are produced in limited amounts during the microorganism–host interaction and may be produced also by the host. Most of the results of the studies reported here must be considered in light of these limitations, despite the attempt to address this point, to put together ecological and available biochemical information.

Nevertheless, there is additional evidence supporting that the hypothesis that BVOCs play a role in transkingdom interactions and are not simply byproducts of bacterial primary or secondary metabolism. This evidence comes from gene regulation studies. On the one hand, it is expected that the production of BVOCs is influenced by chemical–physical parameters, such as nutrient availability, temperature, pH, relative humidity, and the presence or absence of oxygen. On the other hand, recent studies have shown that the production of BVOCs can be stimulated or inhibited during the interaction of bacteria with their hosts consistently with a biological activity of BVOCs in the host [184,185,186,187]. Specifically, in several bacteria, BVOCs production is controlled by GacS/GacA two-component system which, in enteric bacteria and fluorescent pseudomonads, controls primary and energy metabolism and the expression of virulence (secretion systems) or biocontrol factors, including exoenzymes and secondary metabolites (siderophores, antibiotics, BVOCs) [187,188,189].

Other questions still awaiting an answer concern the BVOCs biosynthetic pathways that are, for many BVOCs, still not well-characterized. Here, we attempted to call attention to some biosynthetic pathways of primary importance in the transkingdom interaction between bacteria, insects, and plants, which lead to the production of pyrazines, terpenes and terpenoids, alkanes, and alkenes. We highlighted how some information about the biosynthetic pathways comes from researches in allied fields, such as those investigating the biotechnological exploitation of all these compounds. The elucidation of the biosynthetic pathways underlying BVOCs production will not only will help us better understand the biological role of these compounds, but it will also facilitate their use in various fields, from agriculture to food-processing and food-control, from medicine to veterinary, and from energy production (as biofuels) to environmental management.

## Supplementary Materials

The following are available online at https://www.mdpi.com/2075-4450/10/12/441/s1, Table S1. Insect semiochemicals produced by microorganisms. All molecules that were cited in this review were listed here. Columns: (**A**) IUPAC name of semiochemical compound; (**B**) alternative name of semiochemical compound; (**C**) chemical/biosynthetic class of semiochemical compound; (**D**–**F**) Order (**D**), Family (**E**), Genus and species (**F**) of insect arthropods that are targeted by semiochemical compound; (**G**–**I**) Order (**G**), Family (**H**), Genus and species of microorganisms producing semiochemical (**I**); (**J**,**K**) source of isolation of microorganism (**J**), role of microorganisms in semiochemical metabolism (**K**); (**L**,**M**) general function (**L**) and specific function (**M**) of semiochemical compound; (**N**,**O**) reference number (**N**) and abbreviated reference (**O**); (**P**–**R**) code of compound in KEGG database (hyperlink with KEGG) (**P**), related KEGG pathway map (**Q**), related KEGG pathway name (**R**); (**S**–**U**) chemical class of semiochemical compound as indicated in Pherobase, hyperlinks for information found in Pherobase: compound with behavioral function (**T**) or compound without assigned function (**U**).
